# Skin-protective biological activities of bio-fermented *Aframomum angustifolium* extract by a consortium of microorganisms

**DOI:** 10.3389/fphar.2023.1303198

**Published:** 2023-12-21

**Authors:** Marion Albouy, Simon Aubailly, Olivier Jeanneton, Clarisse Marteau, Lauren Sobilo, Rachid Boulgana, Gerard Bru, Marine Bellanger, Emmanuelle Leblanc, Morgan Dos Santos, Karl Pays, Patrick Choisy, Elodie Bossard, Carine Nizard, Amelie Thepot, Lorene Gourguillon, Anne-Laure Bulteau

**Affiliations:** ^1^ LabSkin Creations, Edouard Herriot Hospital, Lyon, France; ^2^ LVMH Recherche, Saint Jean de Braye, France

**Keywords:** 3D bioprinted skin equivalent, keratinocyte stem cell, aging, fermentation, *Aframomum angustifolium*

## Abstract

**Background:** *Aframomum sp.* is a genus of plants in the Zingiberaceae family. It includes several species, some of which are used in cosmetics for their various properties, making them useful in skincare products, particularly for anti-aging, moisturizing, and brightening the skin. However, to date, there is no experimental evidence on its natural extracts obtained or modified using microorganisms (bio-fermentation) as an anti-aging agent.

**Objective:** The present study aimed to evaluate the antiaging effect of a Bio-fermented *Aframomum angustifolium* (BAA) extract on 3D bioprinted skin equivalent.

**Methods:** The consortium of microorganisms contained *Komagataeibacter, Gluconobacter, Acetobacter, Saccharomyces, Torulaspora, Brettanomyces, Hanseniaspora, Leuconostoc, Lactobacillus, Schizosaccharomyces*. It was developed on a media containing water, sugar, and infused black tea leaves. The seeds of *Aframomum angustifolium* previously grounded were mixed with the culture medium, and the ferments in growth; this fermentation step lasted 10 days. Then, the medium was collected and filtered (0.22 µm) to obtain the BAA extract. To enhance our comprehension of the impact of BAA extract on skin aging, we developed skin equivalents using bio-printing methods with the presence or absence of keratinocyte stem cells (KSC). These skin equivalents were derived from keratinocytes obtained from both a middle-aged donor, with and without KSC. Moreover, we examined the effects of treating the KSC-depleted skin equivalents with Bio-fermented *Aframomum angustifolium* (BAA) extract for 5 days. Skin equivalents containing KSC-depleted keratinocytes exhibited histological characteristics typical of aged skin and were compared to skin equivalents derived from young donors.

**Results:** The BAA extract contained specific organic acids such as lactic, gluconic, succinic acid and polyphenols. KSC-depleted skin equivalents that were treated with BAA extract exhibited higher specular reflection, indicating better hydration of the stratum corneum, higher mitotic activity in the epidermis basal layer, improved dermal-epidermal connectivity, and increased rigidity of the dermal-epidermal junction compared to non-treated KSC-depleted equivalents. BAA extract treatments also resulted in changes at the dermis level, with an increase in total collagen and a decrease in global laxity, suggesting that this extract could help maintain youthful-looking skin.

**Conclusion:** In summary, our findings indicated that BAA extract treatments have pleiotropic beneficial effects on skin equivalents and that the bio-fermentation provides new biological activities to this plant.

## Introduction

Skin aging is characterized by various changes, including wrinkles, uneven pigmentation, dryness, duller microrelief, loss of elasticity, thinning of the epidermis and dermis, and flattened dermal-epidermal junction (Bonte et al., 2019). These changes result from significant modifications in the structure and biology of the skin. The gene expression profile of keratinocytes in the epidermis is altered due to the collapse of the calcium gradient that regulates it, leading to changes in the composition of the cornified envelope, resulting in an impaired barrier function (Rinnerthaler et al., 2015). Hemidesmosomes, which anchor keratinocyte stem cells (KSC) to the dermis at the level of the dermal-epidermal junction (DEJ), are affected by age, leading to a loss of stem cells number (Walko et al., 2015). Key to the age-induced changes affecting hemidesmosomes is the type XVII collagen. It is the least stable hemidesmosome component and, with age as well as with the accumulation of extrinsic damages, its amount decreases (Liu et al., 2019), leading to the gradual emergence of less suitable stem cells resulting in a thinner epidermis. The alteration of hemidesmosomes also affects the DEJ, resulting in weakened substructures that affect the functions of the epidermis and dermis. Its ripples result in dermal papillae that contain capillaries providing nutrient supply to the epidermis (Roig-Rosello and Rousselle, 2020). The dermal papillae also increase the surface area of the DEJ, augmenting the number of hemidesmosomes, strengthening dermal-epidermal connectivity, and keeping both layers well connected. Due to aging, the DEJ flattens, presenting weakened substructures, which affect its functions and those of the epidermis and dermis. The dermis of elderly skin is characterized by decreased thickness, lower synthesis, and degradation of collagen, which leads to increased fragility, loss of density, elasticity, and wrinkles appearance (Haydont et al., 2019).

Understanding the role of KSC in epidermal-dermal crosstalk in aging could lead to strategies to rejuvenate the skin. Indeed, any strategy able to rejuvenate the epidermis or the dermis could have profound impacts on the other skin layer and, therefore, on aging signs.

*Aframomum* is a genus of plants in the Zingiberaceae family that includes several species, some of which are used in cosmetics for their various properties, making them useful in skincare products, particularly for anti-aging, moisturizing, and brightening the skin (Amadi et al., 2016; Niyukuri et al., 2022; Makamwe et al., 2023). However, to date, there is no experimental evidence on its natural extracts obtained or modified using microorganisms (biofermentation) as an anti-aging agent. A Biofermented *Aframomum angustifolium* (BAA) extract was thus developed, fermentation becoming an interesting approach of the cosmetic industries to obtain bioactive molecules-rich ingredients (Majchrzak et al., 2022). To enhance our comprehension of the impact of BAA extract on KSC function and thus in skin aging, we developed skin equivalents using bioprinting methods with the presence or absence KSC. These skin equivalents were derived from keratinocytes obtained from both a middle-aged donor, with and without KSC. Moreover, we examined the effects of treating the KSC-depleted skin equivalents with BAA extract for 5 days. Skin equivalents containing KSC-depleted keratinocytes exhibited histological characteristics typical of aged skin and were compared to skin equivalents derived from young donors. keratinocyte stem cells (KSC). We did not include a control treatment in this study because the objective was to truly assess the impact of this fermented extract on skin stem cells and their anchorage at the dermo-epidermal junction, rather than comparing it to another non-fermented extract.

## Materials and methods

### Plant material and fermentation procedure

Raw *Aframomum angustifolium* seeds, provided by Sotramex (Antananarivo, Madagascar), were milled and reduced into a thin powder at about 500 μm. Before starting the *Aframomum angustifolium* fermentation process, a consortium of microorganisms composed of *Komagataeibacter, Gluconobacter, Acetobacter, Saccharomyces, Torulaspora, Brettanomyces, Hanseniaspora, Leuconostoc, Lactobacillus, Schizosaccharomyces* strains were incubated on a media containing water, sugar, and infused black tea leaves during 3–4 days at 26°C. The grounded seeds of *Aframomum angustifolium* were then added to the resulting mixture at a concentration of 12 g/L and fermentation was carried out for 10 days at a room temperature (about 26°C). The medium was collected and filtered (0.22 µm) to obtain the Bio-fermented *Aframomum angustifolium* (BAA) extract. Non fermented *Aframomum angustifolium* (AA) extract was obtained as previously published (Bonnet-Duquennoy et al., 2007).

### Reagents and standards

Formic acid was purchased from Sigma Aldrich (St. Louis, MO, United States). Deionized water was obtained from a Synergy UV System (Millipore). Ethanol absolute (99.8%), acetonitrile, and methanol were purchased from VWR (Radnor, United States). Phenolic compounds standards including Catechin, Epicatechin, Taxifolin, Hyperoside (kaempferol-3-*O*-galactoside), Isoquercetin (quercetin-3-*O*-glucopyranoside), and Astragalin (kaempferol-3-*O*-glucoside) were purchased from Extrasynthèse; Gallic acid, Rutin (quercetin-3-*O*-rutinoside), Gluconic acid, Quinin acid, Succinic acid, Citric acid, Lactic acid, and Malic acid were purchased from Sigma; Theogallin was purchased from PhytoLab. All other reagents and chemicals used were of analytical grade.

### Chemical characterization and mineral analysis of the bio-fermented *Aframomum angustifolium* (BAA) extract

#### Identification of phenolic compounds

Main phenolics compounds were analyzed using an Agilent 1260 infinity HPLC system coupled to an iQ MSD detector (Agilent Technologies). Chromatographic separation was achieved on an Phenomenex Kinetex ^®^ C18 column (3.0 × 100 mm, 2.6 μm) coupled to an Phenomenex Security Guard™ C18 pre-column (3.0 mm). Samples were carried through the column following a gradient of solvent A (H_2_O; 0.1% formic acid) and B (acetonitrile; 0.1% formic acid). Chromatography was carried out at a flow rate of 0.4 mL min^−1^, using gradient elution according to the program: starting with 5% B for 1 min, reaching 30% B at 15 min, 80% B at 20 min and 100% B at 21 min, holding 100% B for 3 min and coming back to 5% B in 1 min, for a total run time of 25 min. Injection was performed in a full loop mode with 5 μL of the samples. Samples were analyzed in negative ion mode with capillary voltage set at 3.5 kV, nebulizer at 50 psi and dry gas at 11 L min−1, with a dry temperature of 325°C. Fragmentation was performed in Autoacquire MS mode. Phenolic compound identification was performed based on their retention times and extracted ion compared with those of standards.

#### Quantification of organic acids

Quantification of organics acids was achieved on a Phenomenex Hydro-RP column (4.6 × 150 mm, 4 μm). Samples were carried through the column following an isocratic elution mode of solvent A (H_2_O; 0.1% formic acid and at a flow rate of 0.400 mL min^−1^, for a total run time of 25 min. Injection was performed in a full loop mode with 5 μL of the samples. Samples were analyzed in negative ion mode with capillary voltage set at 3.5 kV, nebulizer at 50 psi and dry gas at 11 L. min−1, with a dry temperature of 325°C. Fragmentation was performed in Auto acquire MS mode. Quantification was based on calibration curves obtained using acidified deionized water standard solutions of organic acids. The organic acid content was calculated using the following formula:

$$Organic\;acid\;content\;\left(as\;\%\;of\;BAA\;dry\;matter\right)=\frac{Organic\;acid\;result\;of\;the\;assay\;\left(µg\right)}{BAA\;extract\;assay\;weight\;\left(mg\right)\times BAA\;extract\;dry\;matter\;\%}\times 10$$

#### Mineral analysis

The mineral analysis of the Biofermented *Aframomum angustifolium* (BAA) seed extract was performed at an accredited laboratory (Eurofins, Hamburg, Germany) using ICP-SFMS (Inductively Coupled Plasma-Sector Field Mass Spectrometry) according to SS-EN ISO 17294-2. The cations screened for were Iron, Phosphorus, zinc, Calcium, Potassium, and Sodium.

#### Ethical considerations and human cutaneous cells isolation

Experimental design was done with respect to the ethical permissions. The principal requirements of the Declaration of Helsinki were considered to protect the rights, safety and wellbeing of subjects participating in the study. Before initiating the studies, the investigator had obtained written consent from the participants and full approval from the Freiburg Ethics Commission International for the protocol, protocol amendment(s), if applicable. All participants who provided their skin biopsies for this research provided their written informed consent to participate in this study and for their data to be used for research purposes. Normal human skin samples were obtained from the surgical discard of anonymous healthy patients with informed consent of adult donors or children's parents in accordance with ethical guidelines (French Bioethics law of 2004) and declared to the French research ministry (Declaration no. DC-2021-4346 delivered to LabSkin Creations, Edouard Herriot Hospital, Lyon, France). Approval was not required as per the local legislation where the study was conducted. This study uses strains obtained from deidentified (human samples. Ethical guidelines (French Bioethics law of 2004) did not require the study to be reviewed or approved by an ethics committee because the samples are the surgical discard of anonymous healthy patients.

Primary cultures of young (<5 years) and adult (42 years) normal human epidermal keratinocytes (NHEK) and dermal fibroblasts (NHDF) were established from healthy skin. NHDF subpopulations from papillary (NHDFp) and reticular (NHDFr) dermis were isolated as described (Mine et al., 2008).

#### Epidermal stem cells enrichment and subculture

The strategy of separation of NHEK into populations enriched or depleted in stem cells relied on the differential expression of the transferrin receptor CD71 (Webb et al., 2004). Low level of this marker is associated with KSC, while it expressed at higher levels in differentiating NHEK.

Separation was performed using an anti-human CD71 magnetic microbeads kit (Miltenyi Biotec, Germany) according to the manufacturer’s protocol. Briefly, NHEK were incubated with the beads for 15 min at 4°C. After centrifugation, unbound beads were discarded with the supernatant, while the pellet was resuspended and passed through the column equipped with the magnet. A CD71-depeleted subpopulation of NHEK was recovered in the flow-through. This fraction is enriched in KSC. The CD71-positive keratinocyte population retained on the column, which is depleted in KSC was rinsed and eluted. Both populations were resuspended and numerated to be used for 3D skin tissue engineering.

#### Full-thickness human skin equivalent extrusion-based 3D bioprinting

Bioprinting of skin equivalent samples were produced following the methodology described (Pourchet et al., 2017), partially modified. Yet, we refined the technique by printing layers of NHDFr onto which a layer of NHDFp was added.

The day before printing, the bioink composed of bovine gelatin, low-viscosity alginate and fibrinogen (LabSkin Creations, Lyon, France) was dissolved in calcium-free DMEM (Life Technologies, Carlsbad, United States of America) containing 25 × 10^4^ cells/mL of NHDFp or NHDFr. After incubation under constant agitation for 15 min, the bioink containing cells was loaded into a syringe equipped with a 20 gauges extrusion nozzle. A custom-made bioprinter (LabSkin Creations/TOBECA, Lyon, France), was used to deposit two 1 mm-thick layers of NHDFr and then, on top of it, one 1 mm-thick layer of NHDFp. Bioprinted dermal equivalents were then consolidated by incubating them in a 3% calcium chloride supplemented with thrombin for 1 hour. Once reticulated, bioprinted dermal equivalent containing cohesive distinct engineered papillary and reticular layers were then cultured in LabSkinCULT culture medium supplemented with 10% bovine serum and ascorbic acid and allowed to grow for 7 days at 37°C in 5% CO_2_. The culture medium was changed every 2 days.

After a 7-day period, NHEK were seeded on the surface of the bioprinted dermal equivalent at a density of 25 × 10^4^ cells/cm^2^ to establish a full-thickness skin equivalent. The bioprinted skin equivalent samples were engineered using either CD71-negative NHEK (KSC enriched fraction), or CD71-positive NHEK (KSC depleted fraction) extracted from adult 42-years-old donor or young donor (<5 years) used as a control. After 3 days of culture in immersion, the samples were raised at the air-liquid interface to allow the onset of the differentiation process. After a total of 18 days of total 3D cell culture, bioprinted skin equivalents were harvested and processed for histological, immunohistochemical, atomic force microscopy, and second harmonic generation microscopy analysis. For each cell culture condition and analysis, 3D skin equivalents were produced in triplicate and each experiment was repeated twice.

#### Treatment of full-thickness human skin equivalent depleted of KSC

The bioprinted skin equivalent samples were engineered using CD71-positive NHEK (KSC depleted fraction) extracted from adult 42-years-old donor as described above. After 3 days of culture in immersion, the samples were raised at the air-liquid interface to allow the onset of the differentiation process. KSC- depleted models were then treated, with 0. 1% BAA extract diluted in the culture medium, twice a day. After a total of 18 days of total 3D cell culture, bioprinted skin equivalents were harvested and processed for histological, immunohistochemical, atomic force microscopy, and second harmonic generation microscopy analysis. For each cell culture condition and analysis, 3D skin equivalents were produced in triplicate and each experiment was repeated twice.

#### Histological and immunohistological analysis

Paraffin-embedded formalin-fixed samples were cut into 5 μm sections. After dewaxing and rehydration, sections were stained with haematoxylin, phloxin and saffron (HPS) for routine histological analysis.

Immunofluorescence staining of paraffin sections was performed to detect Ki67, integrin alpha-6, type VII and XVII collagen. 5-μm paraffin-embedded sections were deparaffinized and rehydrated. After heat unmasking, the sections were incubated with a solution of 5% BSA to block nonspecific binding. Sections were then incubated overnight with the primary antibodies at 4°C overnight, followed by an AlexaFluor-568 secondary anti-mouse or anti-rabbit antibodies (Molecular Probes, Invitrogen) for 1 h at room temperature. Nuclear counterstaining using Hoechst 33342 fluorescent dye (Thermo Fisher Scientific, MA, United States) was carried out routinely.

#### Image acquisition and analysis

Specimens stained in HPS were observed using an Axio observer D1 optical microscope (Zeiss, Germany), and images were captured using DS-Ri1 CCD camera (Nikon) and NIS-Elements software (Nikon). Sixteen-bit images were saved in an uncompressed tagged image file format (tiff). Six representative images were captured for each condition in the same manner. Image processing and analysis were performed using the software MBF_ImageJ for microscopy. The parameters in which we were interested were the epidermal thickness for HPS- stained sections, Ki67 nucleus positive cells and the positive surface area of integrin alpha-6 and type VII and XVII collagens.

Epidermal thickness was obtained with a Euclidean distance map (Dos Santos et al., 2015). Pixels corresponding to the epidermis were selected from other pixels. Images were converted to 8-bit binary image. Images corresponding to the area of interest were converted to a 16-bit distance map. To each epidermis pixel (nonzero) in the distance map binary image a value equal to its distance from the nearest background pixel (zero) was assigned. The epidermis basal line was selected and then applied on the distance map. The mean intensity of the basal line corresponds to the mean distance between the basal line and the stratum corneum. Data are expressed in μm.

Ki-67 positive epidermal cells were automatically detected and separated from the background and negatively stained cells by watershed segmentation. Data are expressed in number of positive cells per epidermal field.

Surface positively stained-tissue areas were automatically detected and segmented from other pixels. Images were then converted in binary images, treated by mathematical morphology and sieved for isolating the regions of interest. The surface area of interest was measured automatically. Data were normalized by the length of the epidermal basement membrane, or the dermal surface area and results were expressed in pixel (Rinnerthaler et al., 2015) per μm.

#### Brightness measurement of the skin equivalent surface

The brightness from the surface of the bioprinted skin equivalents was quantified using the Spec. Vol parameter from the GonioLux instrument (Orion Concept, France). This parameter corresponds to the fraction from the incident light reaching the sample with an angle of 30° that is reflected symmetrically by the outermost surface of the material. In the case of the skin, this parameter relates to the hydration level of the *stratum corneum*.

If the GonioLux is frequently used in clinical studies to analyze the skin surface of subjects, its use to study *in vitro* skin equivalents required some adaptations. The device was placed upside down, the probe upward, and skin equivalents were placed on the top, avoiding undesired pressure while ensuring good contact and stability of the sample. Due to the uneven relief of the skin equivalent disks, acquisitions were performed under four different angles (0°, 90° 180°, and 270°). Four acquisitions were performed at each angle, repositioning the skin equivalent each time, and using the triplicate bioprinted skin equivalents of the different experimental conditions.

#### Second harmonic generation imaging

Analyses were performed using a LSM780 inverted confocal microscope (Zeiss, Germany) equipped with a 40x “C-Apochromat” 40x/1.1 M27 objective (Zeiss, Germany). The observation window was 350 μm large, defined by 1024 × 1024 pixels with a resolution of 0.69 × 0.69 × 3 μm. A pulsed biphoton Chameleon laser (Coherent, CA, United States of America) was used at an excitation wavelength of 900 nm. A filter at 583 nm enabled isolating the fluorescent signal of collagen.

#### Atomic force microscopy (AFM)

AFM analysis of skin equivalents was carried with a Bioscope Resolve AFM system (Bruker, MA, United States of America) mounted on an epifluorescence microscope (DMi8, Leica, Germany). Analysis was performed using the PeakForce^®^ QNM/Fast-Force volume mode and a probe with a theoretical stiffness constant of 0.4N/m and a curvature radius <10 nm. Before any measure, the deflection sensitivity of the probe was measured on Sapphire, and its stiffness constant was calibrated using the thermal noise method. The force matrix was acquired by performing 6,200 measures on a 20 by 20 μm zone, corresponding to an image of 64 × 64 px. The loading force to acquire force curves was 20 nN with a ramp size of 10 µM. The elastic modulus was computed using the BioMeca Analysis software (BioMeca, France).

Skin equivalents were placed in PBS buffer at room temperature. Positioning of the probe at the dermal-epidermal junction level was made possible by an anti-integrin alpha-6 fluorescent immunolabelling.

#### Elasticity analysis

The global elasticity was essayed on skin equivalent disks using the contactless WaveSkin^®^ device (Abdouni et al., 2017; Bonnet et al., 2017). Briefly, a controlled air jet (2 mm diameter, 1.5 bars) was applied perpendicularly to the surface of the material analyzed from a distance of 10 mm. The material’s deformation and its return to the initial state after the air jet was stopped are monitored thanks to a triangulation laser and analyzed by a dedicated software developed using the LabViewTM platform (National Instruments, TX, United States). The analysis focused on the residual deformation: the deformation of the skin equivalents when air-jet stopped, enabling the calculation of the elastic modulus. The lower these values are, the higher is the elasticity. Analysis was performed at three different points of each skin equivalent.

#### Statistical analysis

After checking data distribution using the Shapiro-Wilk test (*p* < 0.05), statistical differences were analyzed using analysis of variance (ANOVA) followed by a Turkey-Kramer test. A statistical difference of *p* < 0.05 was considered significant.

## Results

### Determination of bioactive compounds of bio-fermented *Aframomum angustifolium* extract

The *Aframomum* genus has been used in traditional medicine as a source of bioactive components, such as phenolic compounds, diterpenoids, and arylalkanoids (Amadi et al., 2016) Nevertheless, the number of reports describing bioactive compounds content of *Aframomum* sp. extract still needs to be increased. Considering this limited investigation, the chemical composition of the Bio-fermented *Aframomum angustifolium* extract was investigated. In particular, phenolic compounds and organic acids were analyzed using HPLC-UV-ESI-MS.

**FIGURE 1:**
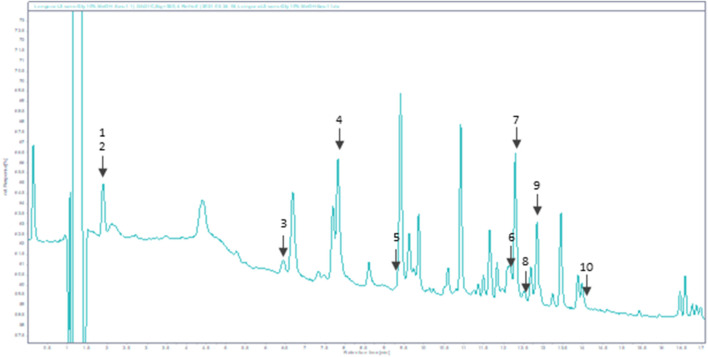
HPLC chromatogram of BAA extract at 320 nm. Peaks: 1-Theogallin; 2-Gallic acid; 3-Catechin; 4-Chlorogenic acid; 5-Epicatechin; 6-Taxifolin; 7-Rutin; 8-Hyperoside; 9-Isoquercetin; 10-Astragalin.

As displayed in Figure 1, Bio-fermented *Aframomum angustifolium* extract was enhanced in molecules. Phenolic acids and glycosylated flavonoids were newly described in *Aframomum angustifolium* seed extract based on their retention times and extracted ions compared with those of standards. The predominant compounds identified were Chlorogenic acid 4), Rutin 7), and Isoquercetin 9) (Figure 1). The BAA extract also contained a smaller amount of flavonoid: Theogallin 1), Catechin 3), Epicatechin 5), Taxifolin 6), Hyperoside 8), Astragalin (10) and phenolic acid: Gallic acid 2).

**FIGURE 2:**
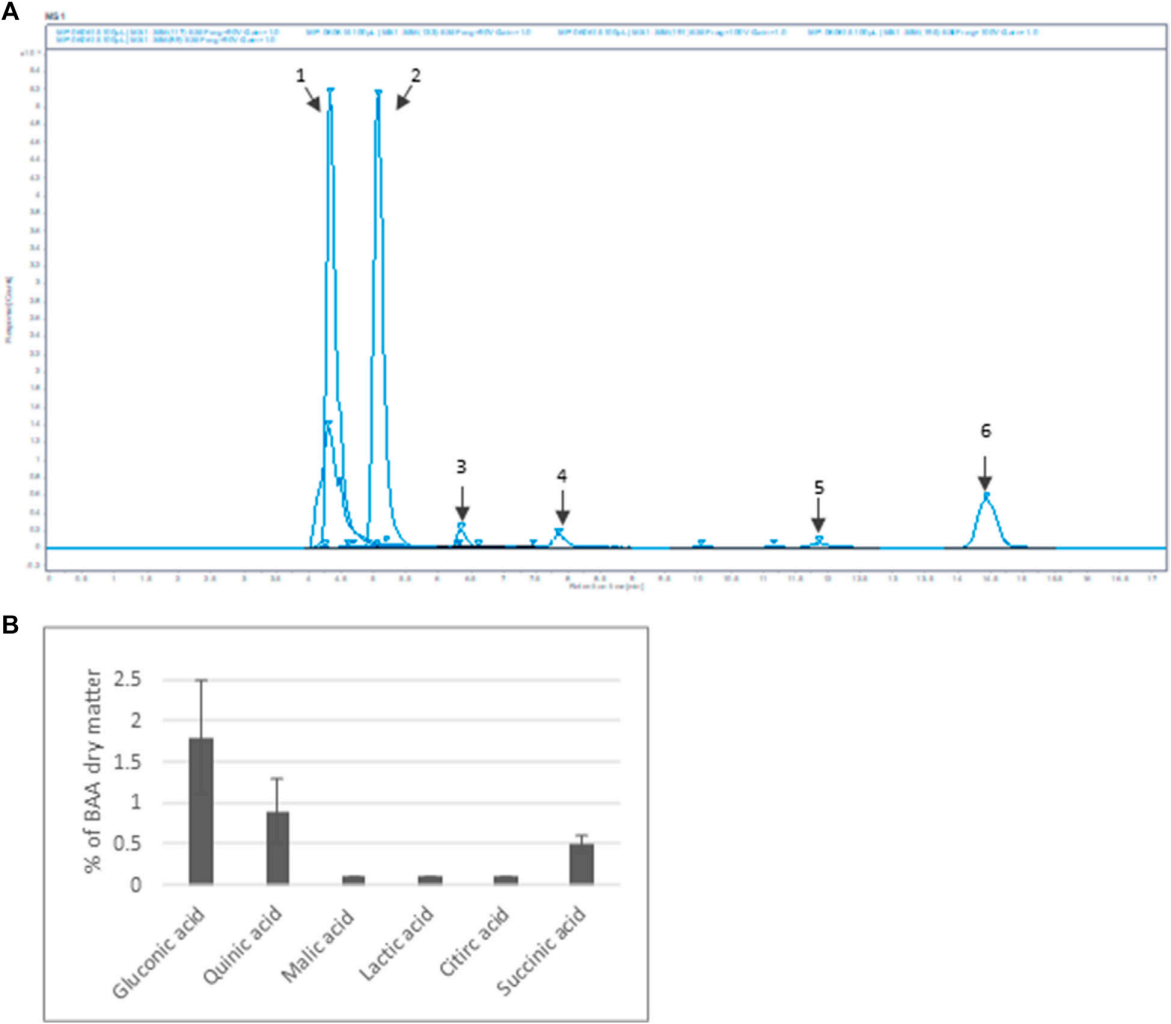
Organic acid content of BAA extract **(A)** MS spectrum. Extracted ion m/z: 1- Gluconic acid; 2-Quinic acid; 3-Malic acid; 4-Lactic acid; 5-Citric acid; 6-Succinic acid **(B)** Quantification of the main organic acids *(n = 3).*

Furthermore, six organic acids were identified and quantified in the Bio-fermented *Aframomum angustifolium* extract (Figures 2A, B). The obtained results, expressed in % of dry weight of BAA extract, are presented in Figure 2B. As described in the literature, fermentation increases the molecular content diversity of plant extract (Majchrzak et al., 2022) The high-molecular compounds are digested by micro-organisms, resulting in an accumulation of low-molecular structures as organic acids in the extract. The main organic acids in the BAA extract were Gluconic acid 1), Quinic acid 2), and Succinic acid 6), with a concentration of about 2%, 1%, and 0.5%, respectively. A slight amount of Malic acid 3), Lactic acid 4), and Citric acid 5) at about 0.1% was measured.

In addition, the mineral composition of the BAA extract was investigated. Interestingly, a high quantity of Calcium, about 68 mg/kg of BAA extract, was measured. Besides, Potassium, Sodium, and Phosphorus were also quantified at 35, 25, and 35 mg/kg, respectively, of BAA extract. Finally, little amount of Iron and zinc were found, around 2.2 and 3 mg/kg of BAA extract.

### Bio-fermented Aframomum angustifolium treatment is influencing the thickness of both the epidermis and dermis

**FIGURE 3:**
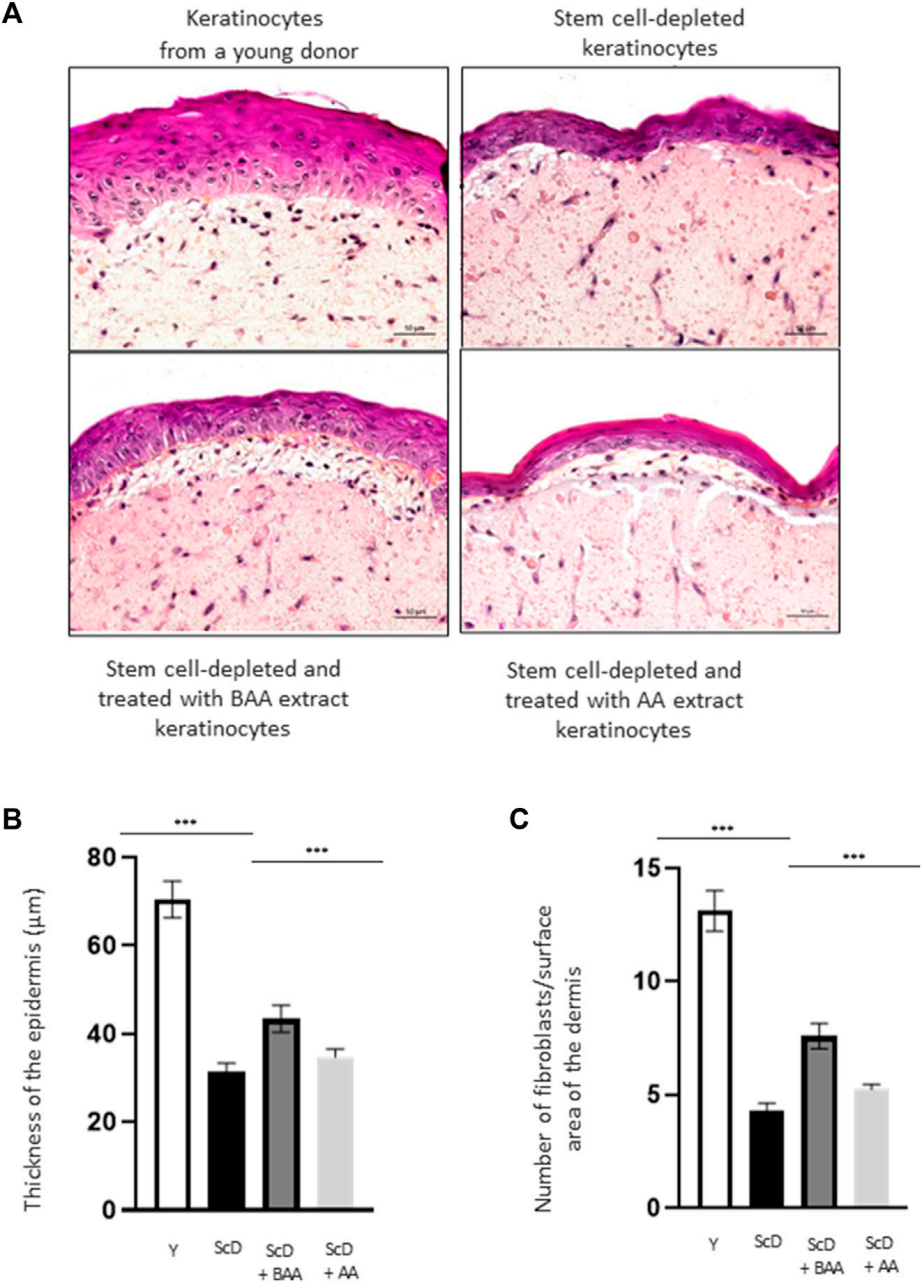
Impact of BAA extract on the morphological study of bioprinted skin models containing young KSC or depleted from KSC. Hematoxylin and Eosin staining allows the observation of RHE morphology: Hematoxylin stains in blue/purple basophilic structures such as the nuclei and Eosin stains in pink acidophilic structures such as the cytoplasm. Histological analysis of skin equivalent. **(A)** Representative histochemical images–scale bar: 50 µm. **(B)** Epidermis thickness. Y: keratinocytes containing young KSC from a young donor, ScD: keratinocytes depleted of KSC from an old donor ScD + BAA: keratinocytes depleted of KSC from an old donor but treated with BAA extract, ScD + AA: keratinocytes depleted of KSC from an old donor but treated with AA extract (non fermented extract), **(C)** Number of fibroblasts. Statistical significance was determined by one-way ANOVA followed by a *post hoc* test (Tukey’s test) *p < 0.05 **p < 0.01 and ***p < 0.001.

To directly analyze the role of BAA extract on skin architecture and functions, we bio-printed skin equivalents having the very same dermal composition but differing in their keratinocytes populations: either very young keratinocytes or keratinocytes from a middle-aged donor that were depleted in KSC to mimic an old skin model. As an initial step to our study, we analyzed the histologic morphology of the bioprinted skin equivalents. To do so, we compared skin equivalents reconstructed with keratinocyte preparations from the middle-aged donor but depleted in KSC (CD71-enriched keratinocyte preparation) or depleted in KSC and treated with BAA extract. We also treated the depleted KSC model with a natural mixture of Aframomum angustifolium seed extract (AA) at the same concentration. Results were compared to those of skin equivalents that received keratinocytes isolated from a young donor. Figure 3A displays representative histological images. The most striking difference between skin equivalents (depleted *versus* young donor) lied in the epidermis, especially its thickness (Figure 3B) but also dermis thickness (Figure 3C). Skin equivalent made with keratinocytes population depleted in KSC presented a very thin epidermis with a hardly visible basal layer. Yet, they are fully differentiated as a *stratum corneum* is present. When skin equivalents were bio-printed with keratinocytes enriched in KSC but treated with BAA extract, the epidermis was significantly thicker, with basal cells presented a characteristic palisade shape. When keratinocytes from a young donor were used as control, the epidermis thickness reached a maximum and presented full differentiation with a clearly visible *stratum corneum*.

The different skin equivalents also presented differences at the level of the dermis. When bio-printed with young keratinocytes, numerous fibroblasts (Figure 3C) and a dense network of extracellular matrix are visible. On the contrary, skin equivalents reconstructed with middle-aged keratinocyte preparation depleted in KSC presented few fibroblasts and a poor extracellular matrix. When a KSC depleted and treated with BAA preparation is used, an intermediate phenotype indicating that BAA extract plays a role not only not only in keratinocytes proliferation and differentiation but also in dermis formation. When employing a KSC that has been depleted and treated with AA extract, the resulting skin thickness is reduced compared to using the fermented extract (Figures 3A–C). Additionally, the proliferation and differentiation of keratinocytes and fibroblasts are less efficient. This suggests that beneficial compounds, previously confined within the substrate structure, are liberated during fermentation, making them more easily accessible and absorbable by the cells. Consequently, our study concentrates specifically on examining the influence of BAA on stem cell function.

### Bio-fermented *Aframomum angustifolium* extract contributes to the reflection of light on the skin’s surface

**FIGURE 4:**
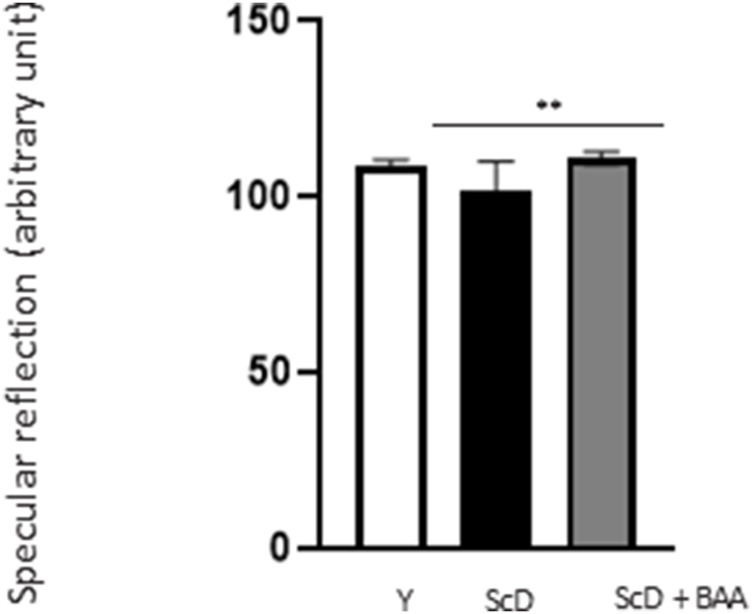
Effect of BAA extract treatment on the specular reflection of the surface of bioprinted skin models containing young KSC or depleted from KSC. Goniolux analysis of the light specular reflection on bioprinted skin. Y: keratinocytes containing young KSC from a young donor, ScD: keratinocytes depleted of KSC from an old donor, ScD + BAA: keratinocytes depleted of KSC from an old donor but treated with BAA extract. Four numerical values were obtained per sample, 3 bioprinted samples per condition. Results are the mean of 12 measurements per conditions. Statistical significance was determined by one-way ANOVA followed by a *post hoc* test (Tukey’s test) *p < 0.05 **p < 0.01 and ***p < 0.001.

Because epidermis thickness and differentiation can play a role in light reflection, we observed their surfaces by studying their specular reflection that depends on the hydration status of the *stratum corneum*. Our results (Figure 4) indicated that skin equivalents made from depleted and treated KSC preparation showed higher specular reflection than those bio-printed with a KSC-depleted keratinocyte preparation indicating that BAA extract can also play a role on skin luminosity.

### Bio-fermented *Aframomum angustifolium* extract is responsible for the proliferation (growth and multiplication) of the epidermis

**FIGURE 5:**
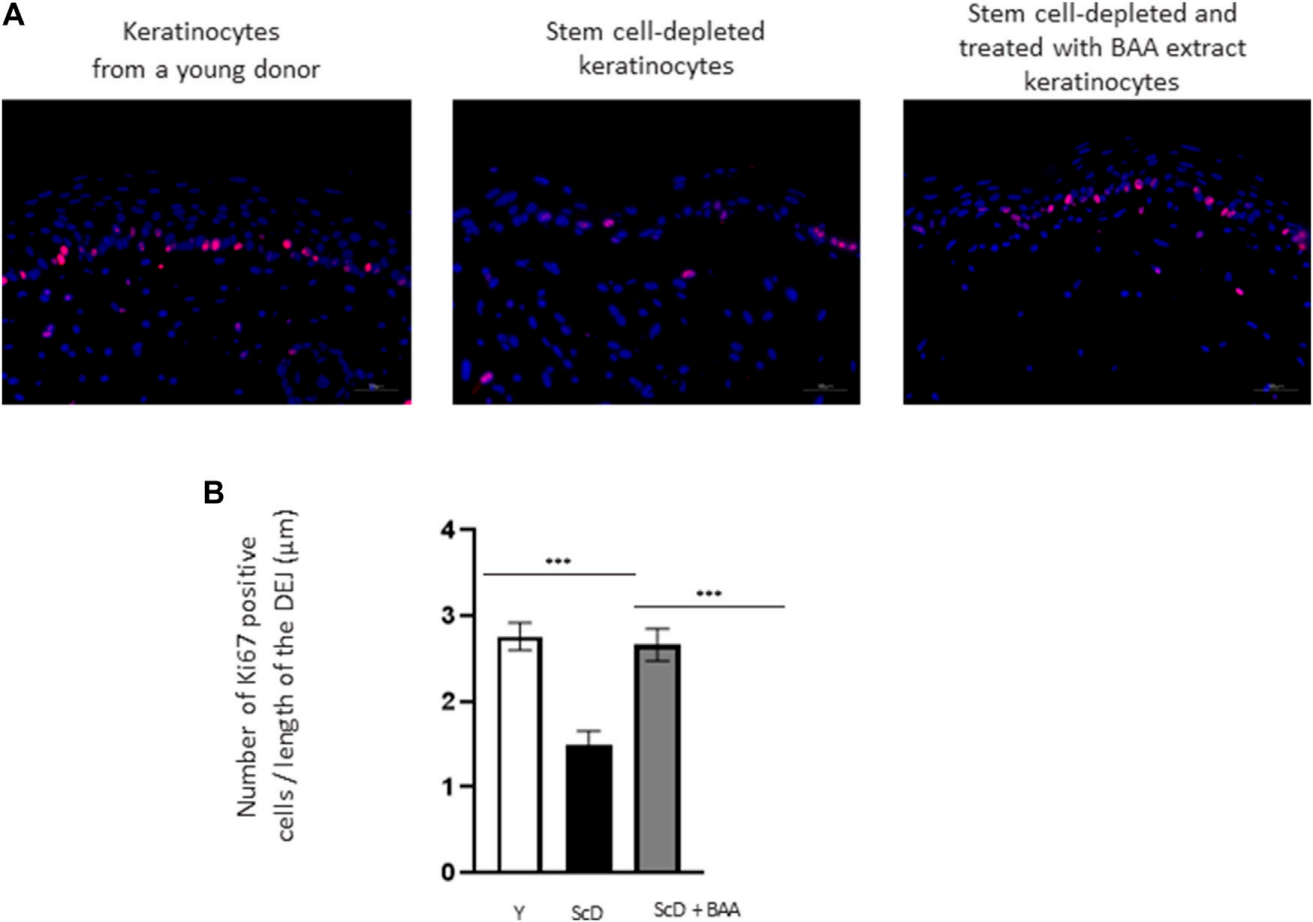
Effect of BAA treatment on cell proliferation in bioprinted skin models containing young KSC or depleted from KSC. **(A)** Immunostaining of Ki67 in bioprinted skin models. **(B)** Quantification of the immunohistochemical labeling of Ki67. Y: keratinocytes containing young KSC from a young donor, ScD: keratinocytes depleted of KSC from an old donor, ScD + BAA: keratinocytes depleted of KSC from an old donor but treated with BAA extract. Analysis was performed using three bioprinted models per conditions as described in the materials and Methods section. Statistical significance was determined by one-way ANOVA followed by a *post hoc* test (Tukey’s test) *p < 0.05 **p < 0.01 and ***p < 0.001.

To better explain the morphological difference observed at the epidermis level, the mitotic activity of keratinocytes from the various skin equivalents was investigated by performing immunohistochemical labeling of Ki67, a marker specific for dividing cells. As expected, Ki67 labeling was essentially visible in the immediate vicinity of the DEJ, confirming the presence of a well-organized layer of KSC. Quantification of the number of Ki67 positive cells over the length of the JDE (Figure 5) showed no significant differences in Ki67 labeling in skin equivalents reconstructed with foreskin keratinocytes or KSC-depleted and treated preparation. Only skin equivalents made from a KSC-depleted preparation showed significant lower Ki67 labeling.

### The extract of bio-fermented *Aframomum angustifolium* stimulates the synthesis of anchorage proteins and facilitates the formation of the dermal-epidermal junction (DEJ)

**FIGURE 6:**
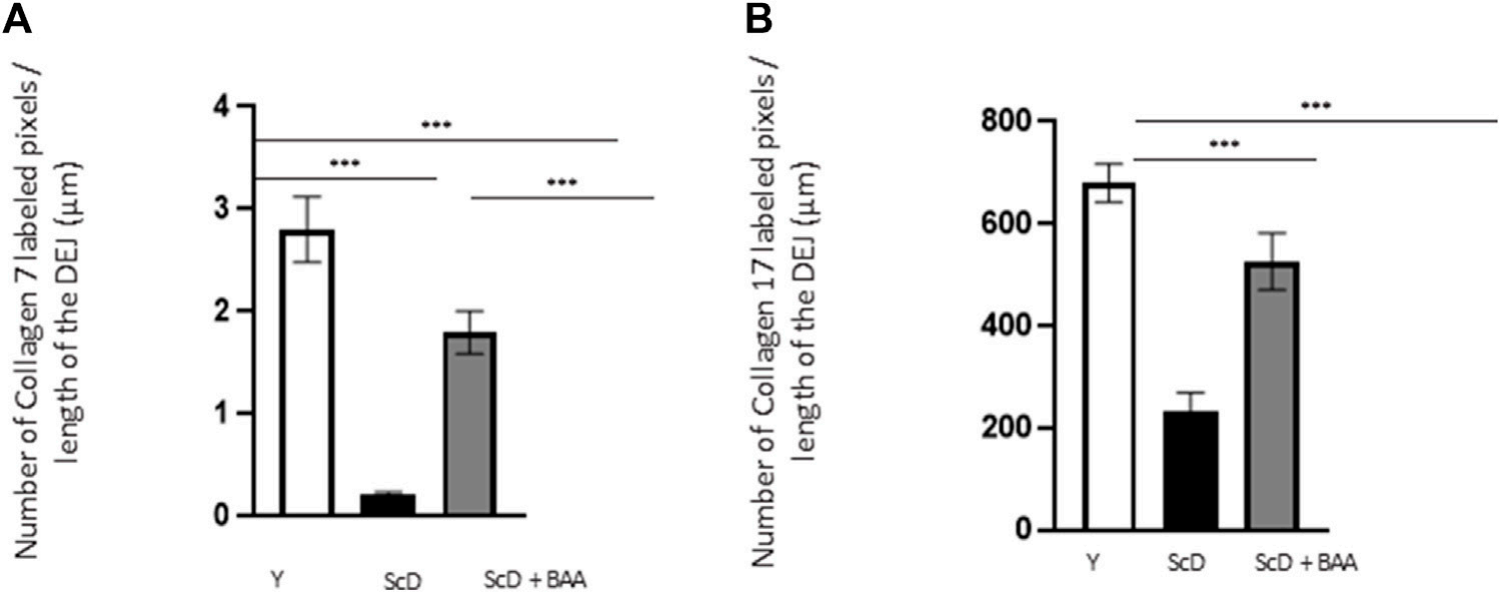
Effect of BAA treatment on collagens synthesis at the dermal-epidermal junction in bioprinted skin models containing young KSC or depleted from KSC. Quantification of the immunohistochemical labeling of the dermal-epidermal junction. **(A)** Collagen VII labeling. Y: keratinocytes containing young KSC from a young donor, ScD: keratinocytes depleted of KSC from an old donor, ScD + BAA: keratinocytes depleted of KSC from an old donor but treated with BAA extract. **(B)** Collagen XVII labeling. Analysis was performed using three bioprinted models per conditions as described in the materials and Methods section. Statistical significance was determined by one-way ANOVA followed by a *post hoc* test (Tukey’s test) *p < 0.05 **p < 0.01 and ***p < 0.001.

**FIGURE 7:**
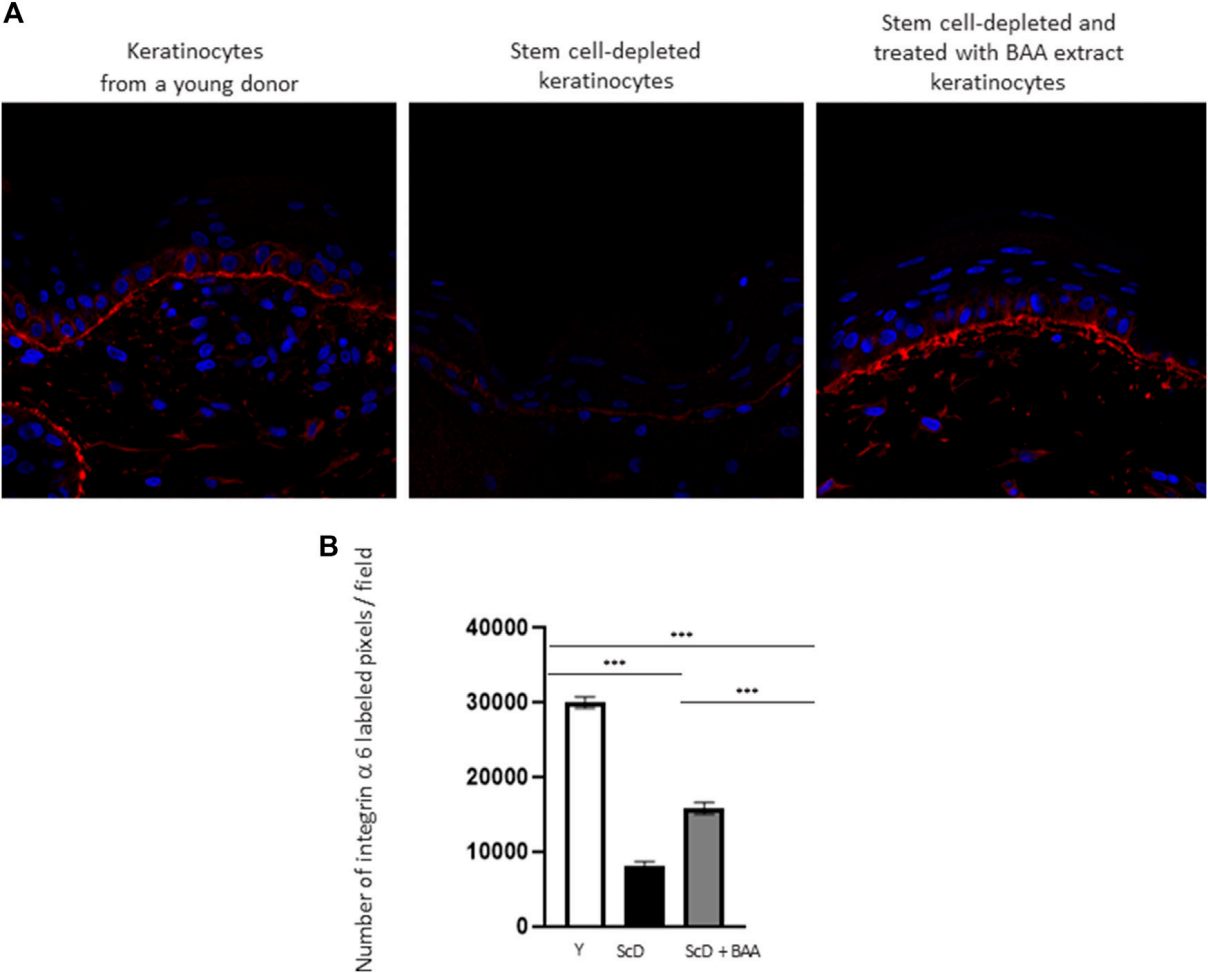
Effect of BAA treatment on integrin α6 labeling at the dermal-epidermal junction in bioprinted skin models containing young KSC or depleted from KSC. **(A)** Immunostaining of integrin α6 in bioprinted skin models. **(B)** Quantification of the immunohistochemical labeling of integrin α6. Analysis was performed using three bioprinted models per conditions as described in the materials and Methods section. Y: keratinocytes containing young KSC from a young donor, ScD: keratinocytes depleted of KSC from an old donor, ScD + BAA: keratinocytes depleted of KSC from an old donor but treated with BAA extract. Statistical significance was determined by one-way ANOVA followed by a *post hoc* test (Tukey’s test) *p < 0.05 **p < 0.01 and ***p < 0.001.

To investigate the role of KSC in DEJ formation, we followed collagen VII, the first collagen synthesized at this level in the bio-printed skin equivalents, and integrin α6, a protein of the hemidesmosome complex. In all skin equivalents, collagen VII labeling was visible, linear, and well organized indicating that the DEJ was mature. Quantification of the number of pixels labeled by length units of the DEJ (Figure 6A) showed a clearly higher level of collagen VII in skin equivalents made with keratinocytes isolated from young donor. Collagen VII expression was highly reduced in the other skin equivalents using KSC-depleted but significantly higher in treated-depleted preparations indicating the importance of BAA extract in collagen VII synthesis which is responsible for DEJ formation. Quantification of collagen XVII (Figure 6B), a transmembrane protein constituting hemidesmosomes (HDs), showed a clearly higher level of collagen XVII in skin equivalents made with keratinocytes isolated from young donor. Collagen XVII expression was importantly reduced in the skin equivalents using KSC-depleted preparations and significantly increased in treated -depleted preparations indicating the importance of BAA extract in collagen XVII and thus in the interactions of stem cells with surrounding cells and the matrix to regulate skin homeostasis. Localization of integrin α6 labeling was essentially restricted to the basal cell layer of the epidermis in all skin equivalents. Nevertheless, significant differences existed upon quantification of this labeling (Figure 7). Skin equivalents made with young keratinocytes showed the highest levels, while those from a KSC-depleted preparation presented the lowest labeling indicating that BAA extract are important for cellular anchorage formation. Indeed, hemidesmosome formation requires calcium which is very concentrated in this extract. (Roig-Rosello and Rousselle, 2020).

### The mechanical properties of the junction between the dermis and epidermis are influenced by the treatment with bio-fermented *Aframomum angustifolium* extract

**FIGURE 8:**
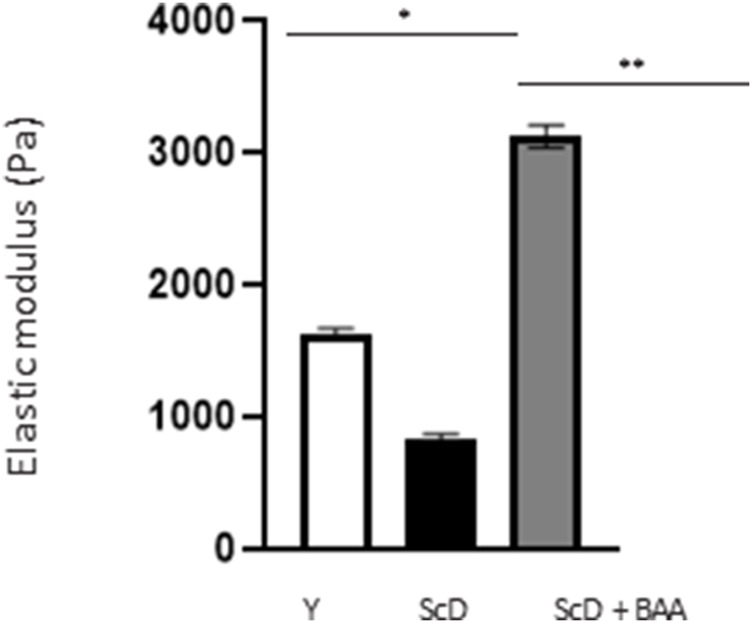
Effect of BAA treatment on average elastic modulus of the dermal-epidermal junction of skin equivalents bioprinted skin models containing young KSC or depleted from KSC. AFM analysis of skin equivalents was carried with a Bioscope Resolve AFM system (Bruker, MA, United States) mounted on an epifluorescence microscope (DMi8, Leica, Germany). Skin equivalents were placed in PBS buffer at room temperature. Positioning of the probe at the dermal-epidermal junction level was made possible by an anti-integrin alpha-6 fluorescent immunolabelling. Analysis was performed using three bioprinted models per conditions. Y: keratinocytes containing young KSC from a young donor, ScD: keratinocytes depleted of KSC from an old donor, ScD + BAA: keratinocytes depleted of KSC from an old donor but treated with BAA extract. Statistical significance was determined by one-way ANOVA followed by a *post hoc* test (Tukey’s test) *p < 0.05 **p < 0.01 and ***p < 0.001.

The differences we identified at the DEJ led us to investigate its mechanical properties in the various skin equivalents. Therefore, we studied its elastic modulus by AFM to construct a force matrix of DEJ regions. Force matrixes revealed that when keratinocytes depleted of KSC are used for bio-printing, the DEJ appears thick. The 2D map showed that the elastic modulus along the DEJ tended to be inhomogeneous, presenting patches of intermediate values in a generally poorly rigid environment. On the contrary, skin equivalents that received young keratinocytes or a KSC-depleted and treated population had a thinner DEJ which force matrix 2D map presented higher elastic modulus with little inhomogeneity. A statistical analysis of all elastic modulus values within the DEJ (Figure 8) indicated that the highest values are obtained when a KSC depleted and treated population is used.

### Bio-fermented *Aframomum angustifolium* extract can induce the synthesis of collagen in the dermis

To apprehend the effect of BAA extract on the dermis, we monitored the fate of the major component of the extracellular matrix: collagen. These experimental series were performed using second harmonic generation (SHG) microscopy.

**FIGURE 9:**
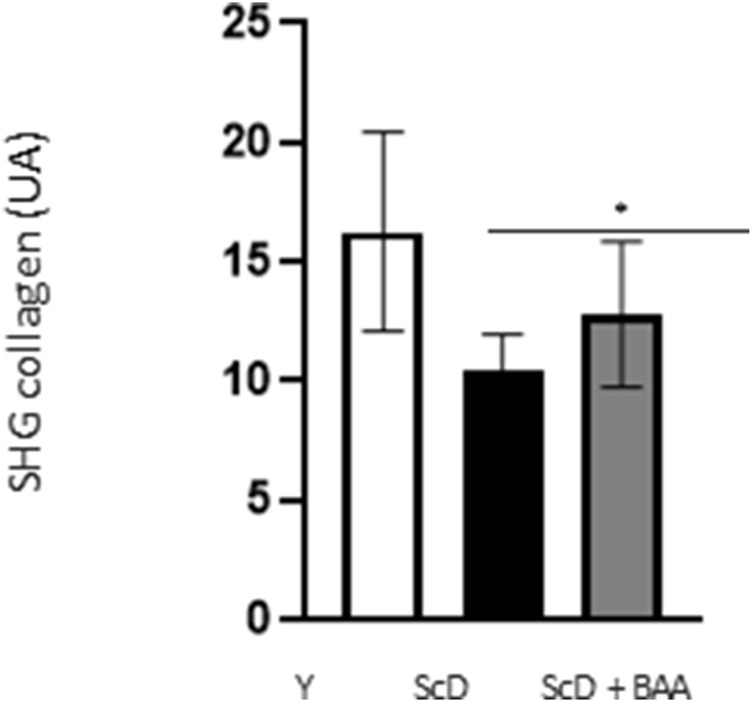
Effect of BAA treatment on total collagen amount in the dermis of skin equivalents bioprinted skin models containing young KSC or depleted from KSC. Analyses were performed using a LSM780 inverted confocal microscope (Zeiss, Germany) equipped with a 40x “C-Apochromat” 40x/1.1 M27 objective (Zeiss, Germany) as described in the Materials and Methods section. Analysis was performed using three bioprinted models per conditions. Y: keratinocytes containing young KSC from a young donor, ScD: keratinocytes depleted of KSC from an old donor, ScD + BAA: keratinocytes depleted of KSC from an old donor but treated with BAA extract. Statistical significance was determined by one-way ANOVA followed by a *post hoc* test (Tukey’s test) *p < 0.05 **p < 0.01 and ***p < 0.001.

Quantification of the SHG collagen signal (Figure 9) indicated that skin equivalent reconstructed from a keratinocyte preparation depleted in KSC presented the lowest level of collagen. The signal was significantly higher when keratinocytes depleted treated in KSC were used.

### Bio-fermented *Aframomum angustifolium* extract contributes to the maintenance of skin elasticity

Finally, in a last experimental series, we investigated the global elasticity and firmness of the skin equivalents. This aspect was analyzed by monitoring the surface deformation of skin equivalents when a calibrated air-jet applied to it stops.

**FIGURE 10:**
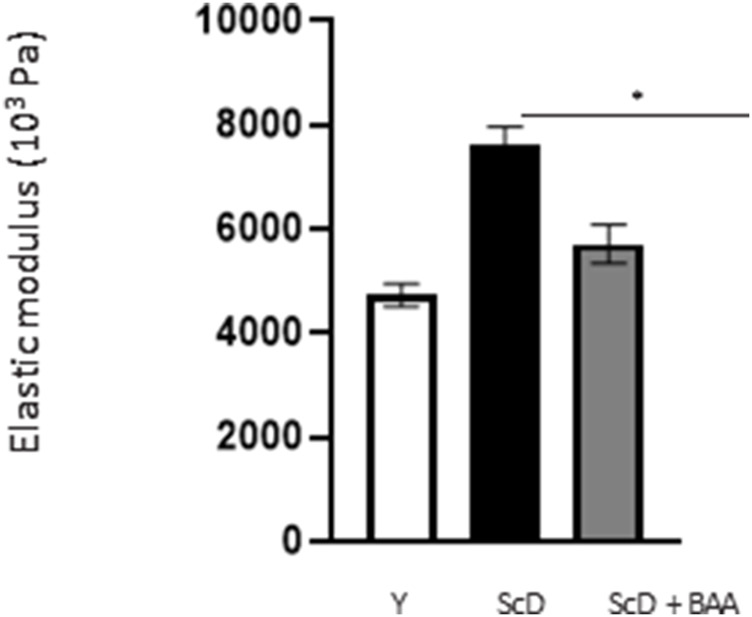
Effect of BAA treatment on cell proliferation on global elasticity of bioprinted skin models containing young KSC or depleted from KSC. The global elasticity was essayed on skin equivalent disks using the contactless WaveSkin^®^ device as described in the Materials and Methods section. Briefly, a controlled air was applied perpendicularly to the surface of the material analyzed. The material’s deformation and its return to the initial state after the air jet was stopped are monitored and the analysis focused on the residual deformation: the deformation of the skin equivalents when air-jet stopped, enabling the calculation of the elastic modulus. Analysis was performed at three different points of each skin equivalent, 3 bioprinted samples per condition. Y: keratinocytes containing young KSC from a young donor, ScD: keratinocytes depleted of KSC from an old donor, ScD + BAA: keratinocytes depleted of KSC from an old donor but treated with BAA extract. Statistical significance was determined by one-way ANOVA followed by a *post hoc* test (Tukey’s test) *p < 0.05 **p < 0.01 and ***p < 0.001.

The computed elastic modulus (Figure 10) indicated significantly higher firmness for skin equivalents reconstructed with keratinocytes depleted from KSC compared to those bio-printed with a KSC-depleted treated preparation which, therefore, showed higher elasticity. Skin equivalents with foreskin keratinocytes presented intermediate characteristics not significantly different from the two other types of skin equivalent constructs.

## Discussion

The data of our study collectively show distinct results for each type of skin equivalent. Such different outcomes are obvious upon histological analysis of the epidermis (Figure 3). Skin equivalents with very young keratinocytes were used as control and presented all the features of young skin: a mature *stratum corneum*, a thick epidermis, a well-organized and dense layer of basal keratinocytes. On the contrary, skin equivalents bio-printed with keratinocytes depleted in KSC have nearly all characteristics of aged epidermis: a reduced thickness with a poor basal layer of stem cells. Treatment with BAA extract enabled the partial restoration of epidermis thickness. Importantly, it led to the establishment of a well-developed and organized stem cell-basal layeThe non-fermented extract did not exhibit the same outcome. Yet, Ki67 immunohistochemical labeling is similar when foreskin and KSC-depleted and treated keratinocytes are used, therefore indicating that the thickness of the epidermis only partially relates to the mitotic activity of KSC (Figure 5).

The analysis of the DEJ revealed differences depending on the fitness and amount of KSC in the various skin equivalents (Figure 6; Figure 7). This is the case for the expression levels of integrin α6 that directly relate to epidermis thickness and for which a BAA treatment led to an increased expression compared to skin equivalents that received a KSC-depleted keratinocyte preparation (Figure 7). The expression of collagen VII follows a similar pattern. These two proteins are essential in ensuring a good dermal-epidermal connectivity: integrin α6 is part of the hemidesmosome complex that collagen VII bridges to dermal collagen fibers (Walko et al., 2015) (Figure 6). This improved connectivity is indeed reflected upon AFM analysis (Figure 8). KSC -depleted and treated models present elastic moduli that are higher and show greater homogeneity than skin equivalent bio-printed with KSC-depleted preparations. This change in mechanical properties could have a profound impact as the epidermis is a mechanosensitive tissue (Evans et al., 2013; Biggs et al., 2020; Roig-Rosello and Rousselle, 2020) (Figure 10). It is already known that changing the stiffness of the substrate onto which KSC grow affects their fate (Trappmann et al., 2012). Therefore, our results indicate that a treatment with BAA extract does not only directly influence epidermis development, it also favorably influences the DEJ towards characteristics resembling those of a young DEJ, which is likely to have numerous beneficial effects.

The increased specular reflection observed for skin equivalents made with depleted and treated preparations could result from such a positive effect (Figure 4). Specular reflection–the incident light reaching the surface of a sample with a defined angle and which is reflected symmetrically–directly relates to the hydration level of the *stratum corneum*. As a matter of fact, specular reflection depends on light scattering at the surface. As increased moisturization decreases light scattering by the *stratum corneum* (Jiang and DeLaCruz, 2011), we can assume that the better DEJ connectivity induced by BAA treatment led to a better nutrient supply and hydration of the epidermis, this could ultimately lead to significant changes in the molecular architecture of the *stratum corneum*, resulting in corneocytes swelling and ultimately leading to improved specular reflection (Mojumdar et al., 2017). From a purely cosmetic point of view, such an effect is of interest as younger skins have more even surface reflectivity (Matsubara, 2012). Besides, dull skin has a significant negative impact on perceived age (Flament et al., 2021).

Another, more unexpected, beneficial effect of BAA treatment is the one we evidenced in the dermis. Compared to skin equivalents made with fibroblasts depleted in KSC, those treated with BAA presented more fibroblasts and a higher amount of total collagen (Figure 9). They also demonstrated a lower skin laxity, as shown by their global elastic modulus (Figure 10). This effect indicates that BAA treatment positively affects dermis organization. We can hypothesize that one of the mechanisms involving nearby interactions by soluble factors could play a role. Among the possible candidates is the double paracrine loop involved in epidermis-dermis homeostasis. In part of this loop, keratinocytes synthesize compounds–interleukin −1 (IL-1) being the main player–that act on fibroblasts to induce their proliferation and migration. This is indeed what we observed. More controversial is the role of this loop in the synthesis of collagen and other ECM components. If a few studies report an enhanced collagen production, similarly to what we obtained, most demonstrate an inhibitory role. Yet, there is a strong agreement that keratinocytes enhance the synthesis of matrix metalloproteases (MMP) by fibroblasts. This suggests an ECM remodeling that is in line with the change in global elastic modulus observed.

Comparing skin equivalents bio-printed with middle-age depleted in KSC and depleted and treated in KSC cells led to rather logical results. However, data obtained from skin equivalents made with foreskin keratinocytes show that very young keratinocytes can lead to different outcomes than depleted in KSC and treated middle-age keratinocytes used. This is the case for specular reflection, the total amount of collagen, and the global elastic modulus. For these three characteristics, skin equivalents made with foreskin keratinocytes show results similar to those made with a KSC-depleted and treated preparation. It is particularly remarkable that foreskin-based alternatives display a well-structured basal epidermal cell layer with strong proliferative abilities and a robust connection between the epidermis and dermis, which is consistent with the characteristics of keratinocytes possessing high stemness potential. Yet, these do not relate to a higher hydration of the *stratum corneum* or any positive effect at the dermis level. These discrepancies clearly indicate differences in the properties of foreskin keratinocytes and KSC-enriched middle-age keratinocytes, whether due to the already aged characteristics of the middle-age keratinocytes or, possibly to the immaturity of foreskin keratinocytes.

The *Aframomum angustifolium* extract obtained by fermentation confirms that this natural transformation process using a consortium of micro-organisms enriched the extract into low-molecular compounds, making fermented extract more biologically active (Majchrzak et al., 2022). The process of fermentation involves the breakdown of substrates, into smaller molecules, thereby enhancing the bioavailability of nutrients in the final fermented product. In essence, beneficial compounds previously “trapped” in the substrate structure are released during fermentation, becoming readily accessible and absorbable by the skin. For instance, fermentation can yield organic acids like malic, fumaric, and citric acids—intermediates in the Krebs cycle—serving as rapid energy substrates for skin-cell mitochondria. Moreover, these acids function as essential mineral (Ca, Mg, Zn, Cu) chelators, consequently mitigating the toxicity of skincare formulations. No specific study directly focuses on stem cells; instead, the research is oriented towards anti-aging effects (Pham et al., 2017; Majchrzak et al., 2022). This study marks the initial exploration of the effects of a fermented plant extract directly on stem cells. Yet, it is important to keep in mind that this work is an initial study of the *Aframomum angustifolium* extract. Therefore, it still suffers from limitations. The first one is inherent to our approach, which is working with a complex extract. Even if we can suspect that organic acids and calcium plays a major role, part of the effects could be due to other compounds, and results could be more intricate than those induced by a purified compound. The second limitation is that all analyses were performed *in vitro*, essentially with isolated cells. It will be essential to test the extract in an appropriate clinical study.

## Data Availability

The raw data supporting the conclusion of this article will be made available by the authors, without undue reservation.
